# Photocatalytic Degradation of Emerging Contaminants

**DOI:** 10.3390/toxics13020080

**Published:** 2025-01-23

**Authors:** Dongyang He, Jiao Qu

**Affiliations:** School of Environment, Northeast Normal University, Changchun 130117, China; hedy443@nenu.edu.cn

## 1. Introduction

Human industrial and domestic activities generate a substantial amount of organic pollutants. Each year, approximately 300 million tons of synthetic chemicals are used globally across industrial and consumer applications, in addition to the agricultural application of 140 million tons of fertilizers and several million tons of pesticides [1]. A large portion of these chemicals enters natural water bodies through multiple pathways, contributing to persistent chemical contamination in aquatic systems, which has become a major global environmental concern. Photocatalytic technology has emerged as one of the most promising strategies to address the contamination of organic pollutants in water. Upon photon absorption by a semiconductor photocatalyst, electron-hole pairs are generated. They can subsequently engage in distinct redox reactions with water and O_2_, facilitating the formation of reactive oxygen species (ROS). These ROS, as potent oxidants, can interact with organic pollutants in water through various reaction pathways, disrupting their molecular structures and thereby facilitating their degradation [2]. In recent years, extensive research has demonstrated the efficacy of photocatalysis in removing various refractory organic pollutants from water, including a wide range of aliphatic alcohols, alkenes, haloalkenes, and others [3]. Furthermore, several developed countries have started exploring the practical implementation of photocatalysis for the removal of organic pollutants in water. For instance, the PSA center in Spain has constructed an industrial-scale photocatalytic wastewater treatment reactor designed to treat industrial effluents containing organic pollutants such as ethylene glycol, phenol, and styrene, with an annual treatment capacity of 1000 m^3^.

However, despite the progress made, several crucial knowledge gaps remain that must be addressed in order to enable further developments in the area of photocatalysis. On one hand, emerging contaminants (ECs), such as pharmaceuticals and personal care products, endocrine disruptors, and pesticides, are widely present in surface water, groundwater, and drinking water. Therefore, researchers should focus on the photocatalytic degradation of ECs, especially since, in practical water treatment processes, no advanced oxidation techniques (including photocatalysis) can completely mineralize organic pollutants. Some ECs, due to their complex molecular structures, may generate a significant amount of by-products during their photocatalytic degradation. These by-products, the long-term effects of which remain largely unknown, can potentially pose a severe health risk to humans. On the other hand, while researchers have continuously developed various high-performance photocatalysts in recent years, it is crucial, from both economic and environmental perspectives, to explore photocatalysts made from elements with abundant natural reserves. Thus, metal-free materials such as black phosphorus and graphitic carbon nitride, known for their low biotoxicity, have been developed to mitigate the risk of secondary pollution [4]. The potential of these materials to advance the photocatalytic degradation of organic pollutants remains largely unrecognized, warranting further investigation. Moreover, the reduction in production costs and the mitigation of hazardous by-products necessitate the development of more sustainable synthesis methods for photocatalysts. Thus, addressing these gaps is of great importance.

## 2. An Overview of Published Articles

Antibiotics, a class of drugs designed to inhibit or eliminate pathogenic microorganisms, have increasingly been recognized as ECs of significant concern. Among these, metronidazole (MNZ), a widely utilized antibiotic belonging to the nitroimidazole class, ranks among the top ten most commonly prescribed medications worldwide. Due to its stable chemical structure, MNZ is difficult to completely remove through conventional treatment methods. In this regard, Shuvo et al. (contribution 1) use a straightforward sol-gel method to synthesize a photocatalyst of Ag and N co-doped SnO_2_ (Ag-N-SnO_2_). Compared to SnO_2_, Ag-N-SnO_2_ exhibits an improved visible-light absorption capacity, a smaller particle size, and a larger specific surface area, which collectively contribute to its superior photocatalytic activity in degrading MNZ. The authors systematically investigate the effects of catalyst dosage, initial MNZ concentration, and solution pH on the photocatalytic degradation efficiency of MNZ, ultimately identifying the optimal reaction condition. Under this condition, the removal efficiency of MNZ reaches an impressive rate of 97.03%. Furthermore, Ag-N-SnO_2_ demonstrates a remarkable activity in achieving MNZ mineralization, as evidenced by a 56% reduction in total organic carbon within 3 h of sunlight irradiation. This work highlights the potential of Ag-N-SnO_2_ as a highly efficient photocatalyst for the remediation of antibiotic contaminants.

Antiviral drugs (ATVs), a specialized class of therapeutic agents designed to treat a wide range of viral infections, have seen a significant increase in production and usage in response to the rising global incidence of viral diseases in recent years. Their widespread application in medical treatments and subsequent discharge into aquatic environments have raised significant concerns regarding their potentially harmful effects on both human health and ecosystems as ECs. Given the limited number of reviews on the photocatalytic removal of ATVs, Zhang et al. (contribution 2) recently publish a comprehensive review. This review provides a detailed summary of the types of ATVs and their contamination status in aquatic environments, outlines the principle of the photocatalytic degradation of ATVs, and discusses the activities, mechanisms, and stabilities of various types of photocatalysts in the degradation of ATVs. More importantly, this review offers a comprehensive discussion of the key challenges in the practical application of photocatalysis for the treatment of ATVs, providing insights for future research and advancing the field.

Over the past few decades, acetaminophen (ACT), a widely used nonsteroidal anti-inflammatory drug, has emerged as one of the most frequently consumed medications worldwide. In this regard, Wang et al. (contribution 3) recently provide a comprehensive summary of the basic properties and consumption patterns of ACT, discussing its contamination in various aquatic environments. This paper highlights the progress made in the photocatalytic removal of ACT, with a particular focus on the degradation activities of different types of photocatalysts, including element-doped catalysts, type-II heterojunctions, and Z-scheme heterojunctions. Finally, the authors outline the challenges associated with the practical application of photocatalysis in ACT treatment, offering valuable insights and guidance for future research in this field.

Currently, most of the photocatalysts that have been studied are primarily metal-based, presenting challenges such as high costs, limited availability, and the potential risk of releasing toxic metal ions into aquatic environments. As a metal-free semiconductor, black phosphorus has garnered significant research interest due to its unique properties of layer-dependent direct bandgap and strong optical absorption. Zhang et al. (contribution 4) are the first to provide a comprehensive review of the development of black phosphorus-based photocatalysts for the photocatalytic degradation of ECs in water. In this article, the authors first explore the fabrication processes of bulk black phosphorus and black phosphorus nanosheets, providing a detailed analysis of the advantages and disadvantages of each method. Subsequently, they categorize black phosphorus-based photocatalysts into heterojunctions and hybrid materials, summarizing their photocatalytic performances against various ECs and the corresponding degradation mechanisms. Finally, they provide insights and future directions for advancing the development of black phosphorus-based photocatalysts in ECs remediation.

In addition to environmentally friendly photocatalyst composition, sustainable synthesis methods for photocatalyst production are equally important, as they help reduce costs and minimize harmful by-products. *Punica granatum* is a widely cultivated plant across the globe, and its extract contains various compounds that can be utilized for capping and reducing nanoparticles. Alshehri et al. (contribution 5) utilize *Punica granatum* extract as an environmentally benign alternative to toxic chemicals, employing it as both capping and reducing agents in the synthesis of ZnFe_2_O_4_ nanoparticles. Subsequently, to further enhance the photocatalytic activity, Cu is deposited onto the ZnFe_2_O_4_ nanoparticles. The authors investigate the photocatalytic activity of the material using Rhodamine B (RhB) as a model pollutant. They examine the effects of catalyst dosage, initial RhB concentration, and solution pH on the photocatalytic degradation efficiency of RhB. Under the optimal condition, a degradation rate of 98.1% for RhB can be achieved. This work offers valuable insights into the utilization of sustainable synthesis methods for the fabrication of efficient photocatalysts for pollutant degradation.

## 3. Future Development Prospects

The papers in this Special Issue offer valuable academic insights, however, a common gap is the lack of research and analysis on by-product toxicity. As the variety of ECs increases, the photocatalytic degradation of these ECs generates a greater number of by-products. Some of these by-products may be more toxic than the parent compounds themselves, thus posing a potential risk to human health. Given the diversity of potential by-products, traditional toxicity testing methods may be limited, as many by-products lack standard reference materials. In this context, quantitative structure-activity relationship (QSAR) analysis offers an effective solution by predicting the acute or chronic toxicity of degradation by-products based on their molecular structures. This method allows for the rapid prediction of toxicity in a large number of degradation by-products, significantly reducing both time and costs. Through QSAR prediction, researchers can identify high-toxicity by-products during photocatalytic degradation, thereby providing guidance for optimizing photocatalytic reaction conditions to minimize their formation.

## List of Contributions

1. Shuvo, M.S.H.; Putul, R.A.; Hossain, K.S.; Masum, S.M.; Molla, M.A.I. Photocatalytic Removal of Metronidazole Antibiotics from Water Using Novel Ag-N-SnO_2_ Nanohybrid Material. *Toxics* **2024**, 12, 36.
2. Zhang, Z.; He, D.; Zhao, S.; Qu, J. Recent Developments in Semiconductor-Based Photocatalytic Degradation of Antiviral Drug Pollutants. *Toxics* **2023**, 11, 692.
3. Wang, Z.; Chen, H.; Rong, C.; Li, A.; Hua, X.; Dong, D.; Liang, D.; Liu, H. Photocatalytic Degradation of Acetaminophen in Aqueous Environments: A Mini Review. *Toxics* **2023**, 11, 604.
4. Zhang, Z.; He, D.; Zhang, K.; Yang, H.; Zhao, S.; Qu, J. Recent Advances in Black Phosphorous-Based Photocatalysts for Degradation of Emerging Contaminants. *Toxics* **2023**, 11, 982.
5. Alshehri, A.; Alharbi, L.; Wani, A.A.; Malik, M.A. Biogenic *Punica granatum* Flower Extract Assisted ZnFe_2_O_4_ and ZnFe_2_O_4_-Cu Composites for Excellent Photocatalytic Degradation of RhB Dye. *Toxics* **2024**, 12, 77.

## Data Availability

Not Applicable.

## References

[1] Schwarzenbach R.P., Escher B.I., Fenner K., Hofstetter T.B., Johnson C.A., Gunten U.V., Wehrli B. (2006). The challenge of micropollutants in aquatic systems. Science.

[2] Wang C.C., Li J.R., Lv X.L., Zhang Y.Q., Guo G. (2014). Photocatalytic organic pollutants degradation in metal-organic frameworks. Energy Environ. Sci..

[3] Mills A., Davies R.H., Worsley D. (1993). Water purification by semiconductor photocatalysis. Chem. Soc. Rev..

[4] Zheng Y., Chen Y., Gao B., Lin B., Wang X. (2020). Black phosphorus and carbon nitride hybrid photocatalysts for photoredox reactions. Adv. Funct. Mater..

